# LED-Fluorescence Microscopy for Diagnosis of Pulmonary Tuberculosis under Programmatic Conditions in India

**DOI:** 10.1371/journal.pone.0075566

**Published:** 2013-10-09

**Authors:** Lord Wasim Reza, Srinath Satyanarayna, Donald A. Enarson, Ajay M. V. Kumar, Karuna Sagili, Sujeet Kumar, Levi Anand Prabhakar, N. M. Devendrappa, Ashish Pandey, Nevin Wilson, Sarabjit Chadha, Badri Thapa, Kuldeep Singh Sachdeva, Mohan P. Kohli

**Affiliations:** 1 International Union against Tuberculosis and Lung Disease(The Union), South-East Asia Regional Office, New Delhi, India; 2 International Union against Tuberculosis and Lung Disease, Paris, France; 3 Central TB Division, Directorate general of health services, Ministry of Health and Family Welfare, New Delhi, India; University of Cape Town, South Africa

## Abstract

**Background:**

Light-emitting diode fluorescence microscopy (LED-FM) has been shown to be more sensitive than conventional bright field microscopy using Ziehl-Neelsen (ZN) stain in detecting sputum smear positive tuberculosis in controlled laboratory conditions. In 2012, Auramine O staining based LED-FM replaced conventional ZN microscopy in 200 designated microscopy centres (DMC) of medical colleges operating in collaboration with India’s Revised National Tuberculosis Control Programme. We aimed to assess the impact of introduction of LED-FM services on sputum smear positive case detection under program conditions.

**Methods:**

This was a before and after comparison study. In 15 randomly selected medical college DMCs, all presumptive TB patients who underwent sputum smear examination in the years 2011 (before LED-FM) and 2012 (after LED-FM) were compared. An additional 15 comparable DMCs that implemented conventional ZN sputum smear microscopy were also selected for comparison between 2011 and 2012.

**Results:**

The proportion of presumptive TB patients (PTP)found sputum smear positive increased by 30%- from 13.6% (3432/25159) in 2011 to 17.8% (4706/26426) in 2012 (P value <0.01) in the sites that implemented LED-FM microscopy, whereas in DMCs where the ZN staining procedure is followed the proportion of sputum smear positive had remained unchanged (13.0%versus 12.6%;P value0.31).

**Conclusion:**

Use of LED-FM significantly increased the proportion of smear positive cases among presumptive TB patients under routine program conditions in high workload laboratories. The study provides operational evidence needed to scale-up the use of LED-FM in similar settings in India and beyond.

## Introduction

Light-Emitting Diode Fluorescence Microscopy (LED-FM) for sputum smear examination is recommended by the World Health Organization (WHO) for detection of acid-fast bacilli in high tuberculosis (TB) burden countries [1]. LED-FM has higher sensitivity (∼8–10%) [2], [3]and similar specificity [4]as compared with Ziehl-Neelsen staining based bright field sputum smear microscopy(ZN SSM) in detecting sputum smear positive TB cases and is more efficient [4], [5].

In India (high TB burden country), [6] medical colleges have tertiary care hospitals functioning in close collaboration with Revised National Tuberculosis Control Programme (RNTCP) for TB care and control. These hospitals have RNTCPs designated microscopy centre (DMC) for the diagnosis of sputum smear positive pulmonary TB using ZN stain based bright field microscopy. In the year 2009/10 and 2010/11, 611683 and 689342 presumptive TB patients (PTP)were examined, out of which 92071 (15.1%) and 95272 (13.8%) were sputum smear positive respectively in 291 out of 321 medical colleges RNTCP DMC’s [7]. However, one has to admit due to high volume of cases for sputum examination at the DMC and inadequate staffing there is often delay in examination of slides and so the diagnosis. In 2012, the South-East Asia office of International Union Against TB and Lung Disease (USEA) in partnership with RNTCP and National Task Force (NTF) took initiation in implementing project LIGHT(Light Emitting Diode Fluorescence Microscopy in Gaining TB Cases in High Work Load TB Hospitals). The project LIGHT involved in replacing existing RNTCP ZN staining Bright field to AO staining LED FM procedure. This project LIGHT was supported by STOP TB partnership’s TB REACH under wave 2 Grant.

The performance of LED-FM in improving yield of sputum smear positive TB cases has not been evaluated under routine programmatic conditions in India and elsewhere in the world. In this study, we assessed whether implementation of LED-FM services in these settings was operationally feasible and led to increased yield of smear positive TB among presumptive TB patients (traditionally referred to as ‘TB suspects’) who underwent sputum smear examination for diagnosis of pulmonary TB.

## Methods

### Study Design

This was a before-and-after study designed to evaluate the impact of introduction and implementation of LED-FM services in detecting sputum smear positive TB cases in medical college DMCs under RNTCP, India.

### Study Setting

There are over 350 medical colleges across India and each of these is associated with tertiary care teaching hospitals. Most of these medical colleges collaborate with RNTCP and have a designated microscopy centre (DMC) for the diagnosis of sputum smear positive pulmonary TB cases and a facility for treatment of TB patients [8], [9]. The DMCs are staffed with trained laboratory technicians(LT) and a senior TB laboratory supervisor (STLS) responsible for ensuring the quality of microscopy [8].

In 2012, ZN SSM was replaced by Auramine O based LED-FM in 200 medical colleges RNTCP DMCs. The training module of RNTCP for fluorescence microscopy was revised by a technical working group consisting members from National TB Reference Laboratories (NRL) and Intermediate TB Reference Laboratories (IRL), Central TB Division (CTD), WHO, FIND, and The Union and was approved by CTD. Based on this module LTs and STLSs were trained for 3 days and 4 days respectively at NRLs and IRL. The training consisted of modular readings, interactive lectures, discussions and hands-on training. The trainees were only certified after they successfully read the panel slides of different grades and scored 70% in the post test based on MCQ’s.

In these DMCs, each PTP undergoes two sputum smear examinations including one ‘spot’ and a second ‘early-morning’ sputum specimens [10]. The sputum smear examination grades were reported by using 400X and 1000X magnification for LED-FM and ZN SSM respectively (**Table 1** **)** [11]. A person with one or both samples positive for acid fast bacillus (any grade), is considered as a case of sputum smear positive pulmonary TB [10]. The results of LED-FM were recorded in existing standardized laboratory registers by the LT, using standard definitions of the RNTCP [12].

**Table 1:** Guidelines for reporting sputum smear results adopted by Revised National TB Control Programme in India.

IUATLD/WHO scale(1000× field = HPF) Grades	Ziehl Neelsen (ZN) staining based bright fieldmicroscopy(1000X magnification: 1length = 2 cm = 100 HPF)	LED Fluorescence based sputum smearmicroscopy (400X Magnification: 1length = 40 fields = 200 HPF)
Negative	Zero AFB/1 length	Zero AFB/1 length
Scanty	1–9 AFB/1 length or 100 HPF	1–19 AFB/1 length
1+ grade	10–99 AFB/1 length or 100 HPF	20–199 AFB/1 length
2+ grade	1–10 AFB/1 HPF on Average	5–50 AFB/1 field on Average
3+ grade	>10 AFB/1 HPF on Average	>50 AFB/1 field on Average

External quality assurance (EQA) protocol for sputum smear microscopy as specified by the RNTCP for ZN microscopy and LED-FM microscopy was in place in all these DMCs. The EQA guidelines related to LED-FM was circulated to the districts implementing LED-FM in medical colleges RNTCP DMC. Onsite evaluation of microscopy (ZN SSM and LED-FM) is routinely carried by STLS on monthly basis. Five positive and five negative slides are checked between two visits of the STLS by a systematic random un-blinded sampling. They are also advised to read the doubtful slides. The LTs were also advised to read the doubtful slides in higher magnification i.e. 1000X for confirmation. The random blinded rechecking (RBRC) is also carried at district level on monthly basis for ZN SSM [13]. For LED-FM one medical college DMC is identified as RBRC sites and 4–8 DMC with LED-FM are linked to RBRC Site. The RBRC is carried out by LED-FM trained STLS under the supervision of District TB Officer. The slides were re-stained before reading to avoid discrepancy in reading. Onsite evaluation (OSE) was also carried by technical consultants of the project in their supervisory visits. It may be noted that no discordances were found between the results of laboratory technician and that of the supervisor during OSE and RBRC.

### Study Population, Sample Size, Sampling Technique

The target population included persons with presumptive TB referred for diagnostic sputum smear examination at the above described DMCs. The results of sputum examinations of persons with presumptive TB attending these DMCs between July to December 2011 using ZN SSM (2011/ZN) were compared with those results between July to December 2012 from LED FM (2012/FM).

In order to assess whether the increase in the proportion of sputum smear positive TB cases were at-least 10% from the baseline (assuming it to be 10% before the introduction of LED-FM services) with 80% power and 95% confidence interval, a design effect of 2, a sample size of 14,950 persons with presumptive TB were required from the 2011 and 2012 periods. As ∼2000 patients with presumptive TB undergo sputum smear examination per DMC per year, in order to achieve the sample size a minimum of 15 DMCs were required. The 15 DMCs were selected randomly by population proportion to size sampling. For each of these 15 DMCs, another DMC within the same district, with comparable workload and implementing ZNSSM services throughout the period was chosen to act as a control group. For this group, ZN SSM results during intervention period (2012/ZN) were compared with ZN SSM results collected during baseline period (2011/ZN).

### Study Variables and Data Sources

The study variables included age, sex and results of each of the sputum samples. Data was extracted from the RNTCP Laboratory Registers maintained at the selected facilities.

### Data Analysis

The data was double-entered into electronic format created in EpiData (Version 3.1, EpiData association, Odense, Denmark) which were further validated and analysed.

The data was cross-tabulated, comparing the period prior and after introduction of LED-FM. Statistical significance of the result was obtained from Pearson’s Chi Square test with Yates correction and P≤0.05 was considered statistically significant.

### Ethics Approval

Ethics approval for the study was obtained from Ethics Advisory Group of International Union Against TB and Lung Disease. Administrative approval to access the records was obtained from Central TB Division, Ministry of Health and Family Welfare, Government of India. This study was record review of laboratory registers and data was analyzed anonymously. The informed consent of the patients was also waived by EAG for this study.

## Results

A total of 25,685 and 27,030 TB symptomatics underwent sputum smear examination in the selected 15 medical colleges during the baseline period (2011/ZN) and intervention period (2012/FM) respectively. Interestingly nearly two-thirds were males. The median age (interquartile range) was 45 (30–60) years for 2011/ZN and median age (interquartile range) was 45 (29–59) years for 2012/FM. The patient population before and after LED-FM implementation was similar by age and sex. Total of 25,159 (2011/ZN) and 26,426 (2012/FM)patients with presumptive TB were available for analysis after excluding the patient with presumptive TB who did not submit either spot or early morning sputum in 2011/ZN (526,2.0%) and 2012/FM (604,2.2%) (**Table 2**). Whereas, in 15 comparable DMCs implementing ZN microscopy, the total number of sputum smear examined were 16,531 (2011/ZN) and 16,403 (2012/ZN) and all were analyzed (Table 2).

**Table 2:** Sputum smear examination results in selected control and medical college (intervention)microscopy centres in India, before (July–December 2011) and after (July–December 2012) introduction of LED-FM.

	Yield of positives				
DMC type	Year/Type†	Examined*	Number	%	P value**
Control DMC	2011/ZN	16,531	2,152	13.0	0.31
	2012/ZN	16,403	2,064	12.6	
MedicalCollege DMC	2011/ZN	25,159	3,432	13.6	<0.01
	2012/LED-FM	26,426	4706	17.8	

* Those who underwent both sputum examinations,

** Chi-square test,

† ZN = Ziehl Neelsen staining microscopy; LED-FM = Light Emitting Diode fluorescence microscopy.

The proportion of sputum smear positive increased from 13.6% (2011/ZN) to 17.8% (2012/FM) (p-value <0.01) after implementation of LED-FM microscopy. The percentage change in the positivity rate was 30% (**Table 3**). The proportion of sputum smear positive in control DMCs (2011/ZN and 2012/ZN) that performed ZN SSM throughout remained unchanged (13%, 2152/16531 versus 12.6%, 2064/16403, p-value 0.31).

**Table 3:** Sputum smear examination results, by age and sex, in selected medical college microscopy centres in India, before (July–December 2011) and after (July–December 2012) introduction of LED-FM.

Characteristic		2011, ZN Microscopy			2012, LED Microscopy			Change			
		Yield of positives			Yield of positives						
		Examined	Number	%	Examined	Number	%	Total	%	(95% CI)	p-value*
**Total**		25,159	3432	13.6	26,426	4,706	17.8	4.2	30	(26–33)	<0.01
**Sex**	**Male**	16,576	2510	15.1	17,435	3,398	19.5	4.4	29	(23–34)	<0.01
	**Female**	8550	918	10.7	8950	1306	14.6	3.9	36	(27–45)	<0.01
	**Unknown**	33	4	12.1	41	2	4.8	−7.3	−60	(−167–47)	0.29
**Age group**	**0–14**	1038	52	5.0	1,109	83	7.5	2.5	49	(9–90)	0.03
	**15–24**	3255	597	18.3	3,535	811	22.9	4.6	25	(15–36)	<0.01
	**25–34**	3785	592	15.6	4,014	780	19.4	3.8	24	(13–35)	<0.01
	**35–44**	4257	668	15.7	4,527	899	19.9	4.2	27	(16–27)	<0.01
	**45–54**	4703	731	15.5	4,869	927	19.0	3.5	23	(13–32)	<0.01
	**55–64**	4218	499	11.8	4,388	749	17.1	5.2	44	(32–57)	<0.01
	**65+**	3535	275	7.8	3,565	449	12.6	4.8	62	(44–80)	<0.01
	**Unknown**	368	18	4.9	419	8	1.9	−3.0	−61	(−113–8)	0.02

* Chi-square test; ZN = Ziehl Neelsen staining microscopy; LED-FM = Light Emitting Diode fluorescence microscopy.

The increase in proportion of sputum smear positive in the sites that implemented LED-FM services was observed across both sexes and all age sub-groups (**Table 3**).However, the percentage change in positivity was higher in extreme of age groups 0–14 years (49%, p-value 0.03) and 65+ years (61%, p-value <0.01). There was an increase across all categories of smear quantification with the use of LED-FM (p-value <0.001) (**Table 4**) with a substantial increase in scanty positives.

**Table 4:** Sputum smear examination results, by smear grading, in selected medical college Designated microscopy centres in India, before (July–December 2011) and after (July–December 2012) introduction of LED-FM.

Highest Grade of patient	ZN Microscopy 2011		LED-FM 2012		Change
	Number	%	Number	%	%
Negatives	21727	86.4	21720	82.2	−4.9
Scanty	377	1.5	777	2.9	93.3
1+ Positive	1036	4.1	1236	4.7	14.6
2+ Positive	714	2.8	886	3.4	21.4
3+ Positive	1305	5.2	1807	6.8	30.8
Total	25159	100	26426	100	

Chi square test = 57.69 (df-3, for positive grades) p-value <0.001; ZN = Ziehl Neelsen staining microscopy; LED-FM = Light Emitting Diode fluorescence microscopy.

## Discussion

Our study is the first to show the successful implementation of LED-FM AO staining services in routine programmatic conditions in India. We found that the introduction of LED-FM services in medical college DMCs resulted in an increase in the yield of pulmonary sputum smear positive pulmonary TB patients. Similar findings have been reported from peripheral laboratories of China [14].The significant increase in the proportion found sputum smear positive was largely due to the inherent advantages of LED-FM, although other contributory factors (such as improvement in the proficiency of the LTs due to additional training they received and new microscopes)may have played a role to a lesser extent.

Previous studies, which were specifically-designed in research settings, showed an increase of 8–10% in the proportion sputum positive patients with LED fluorescence microscopy. These studies had shown that the increase in yield in positivity was predominantly among those with scanty grade[4], [14]–[16]. In line with these studies, the percentage change in scanty grades by LED-FM was 93.3% in comparison to ZN SSM. The studies that re-examined the same location on a smear slide by ZN where AFB had been found with FM indicated that some AFB might no longer be demonstrable [17], [18].The increased sensitivity of FM could attributed to several reasons which include i) stronger absorbability of mycolic acid for carbol-auramine than carbol-fuchsin leading to larger number of acid fast bacilli stained with FM as compared to ZN [19] ii)larger field area examined using high power fields using FM as compared to oil immersion fields by ZN andiii) sharper contrast between bacilli and the background enabling easier identification. The studies in past also showed that quality of commercially available basic fuchsin powder (a mixture of chemicals) used to prepare carbol fuchsin in ZN stain varied considerably which may miss lower grades of smear positivity [20].The unpublished report by Kim KM from China also revealed variation in carbol fuchsin dye content in solution collected from different laboratories located in different places. The quality of commercially available basic fuchsin may miss the lower grades. However, variation of sputum smear grades with different brands of auramine-O as a compound for fluorescence microscopy has not been reported.

There has been a concern expressed in the past about higher chance of false-positive smears (due to impurities in Auramine, food particles and artefacts which produce fluoresce) with LED-FM and the suggestion that all scanty and doubtful cases be confirmed by ZN SSM [21]. However, this only negates the increased sensitivity and the efficiency gains of LED-FM and should be discouraged. Alternate approaches to ensure quality should be deployed including adequate initial training, close supervision, institution of internal quality control mechanisms and external quality assessment using random blinded rechecking followed by retraining [22].

Sputum smear fluorescence microscopy in Senegal revealed that 5.8% of sputum smear positive follow up cases had scanty grades in comparison to new cases (2.5%) [15]. Hence, the implementation of LED-FM in future will also help in management of sputum smear positive follow up cases.

We did not collect information on the number of smear positive TB cases initiated on treatment. This would have been helpful to ascertain if increased number of cases detected actually translated into increased number of cases initiated on treatment. Further, our study evaluated the performance LED-FM services in tertiary care teaching hospitals. There is need for further assessment of performance in other more peripheral microscopy centres to assess the overall expected impact of roll-out of LED-FM services on case notification rates.

This study was limited by the fact that the data were extracted from routine records and we were unable to control the quality of data recording. As an operational research study, it lacked the rigour and internal validity of an intervention trial. The patient populations compared might have had important differences which might be partially responsible for the difference in results. Measuring all the patient characteristics was beyond the scope of the current study and while we are cognizant of this limitation, we note the groups were similar by age and sex and feel the random selection of sites would have mitigated any differences. We evaluated routine practice and have no information concerning skills and knowledge of those undertaking the sputum smear examinations and if they varied between the two time periods. Further studies assessing cost-effectiveness of LED-FM in comparison to ZN microscopy are in progress and will be reported separately.

## Conclusions

The implementation of LED-FM in high work load medical college DMCs increased the detection of smear positive cases. Our study has confirmed the advantage of LED fluorescence microscopy in routine practice in busy laboratories, previously shown in controlled research environments. This provides valuable operational evidence needed for scale-up of this technology in similar settings in India and beyond.
